# Methyl-CpG-binding domain sequencing reveals a prognostic methylation signature in neuroblastoma

**DOI:** 10.18632/oncotarget.6477

**Published:** 2015-12-06

**Authors:** Anneleen Decock, Maté Ongenaert, Robrecht Cannoodt, Kimberly Verniers, Bram De Wilde, Geneviève Laureys, Nadine Van Roy, Ana P. Berbegall, Julie Bienertova-Vasku, Nick Bown, Nathalie Clément, Valérie Combaret, Michelle Haber, Claire Hoyoux, Jayne Murray, Rosa Noguera, Gaelle Pierron, Gudrun Schleiermacher, Johannes H. Schulte, Ray L. Stallings, Deborah A. Tweddle, for the Children's Cancer Leukaemia Group (CCLG), Katleen De Preter, Frank Speleman, Jo Vandesompele

**Affiliations:** ^1^ Center for Medical Genetics, Ghent University, De Pintelaan, Ghent, Belgium; ^2^ Cancer Research Institute Ghent (CRIG), De Pintelaan, Ghent, Belgium; ^3^ Bioinformatics Institute Ghent From Nucleotides to Networks (BIG N2N), De Pintelaan, Ghent, Belgium; ^4^ DAMBI, VIB Inflammation Research Center, Technologiepark, Ghent, Belgium; ^5^ Department of Respiratory Medicine, Ghent University, De Pintelaan, Ghent, Belgium; ^6^ Department of Pediatric Hematology and Oncology, Ghent University Hospital, De Pintelaan, Ghent, Belgium; ^7^ Department of Pathology, Medical School, University of Valencia, and Health Research Institute INCLIVA, Blasco Ibañez, Valencia, Spain; ^8^ Department of Pathological Physiology, Department of Pediatric Oncology, Masaryk University, Černopolní, Brno, Czech Republic; ^9^ Northern Genetics Service, Institute of Genetic Medicine, Central Parkway, Newcastle upon Tyne, United Kingdom; ^10^ Department of Pediatric Oncology, Institut Curie, rue d'Ulm, Paris, France; ^11^ Centre Léon Bérard, Laboratoire de Recherche Translationnelle, rue Laennec, Lyon, France; ^12^ Children's Cancer Institute, Lowy Cancer Research Centre, UNSW, Randwick NSW, Australia; ^13^ Pediatric Hemato-oncology, CHR Citadelle, Liège, Belgium; ^14^ Unité de Génétique Somatique, Institut Curie, rue d'Ulm, Paris, France; ^15^ U830 INSERM, Recherche Translationelle en Oncologie Pédiatrique (RTOP) and Department of Pediatric Oncology, Institut Curie, rue d'Ulm, Paris, France; ^16^ Department of Pediatric Oncology and Hematology, University Children's Hospital Essen, HufelandstraΔe, Essen, Germany; ^17^ National Children's Research Centre, Our Lady's Children's Hospital, Crumlin, Dublin, Ireland; ^18^ Department of Molecular and Cellular Therapeutics, Royal College of Surgeons in Ireland, York House, Dublin, Ireland; ^19^ Newcastle Cancer Centre, Northern Institute for Cancer Research, Newcastle University, Framlington Place, Newcastle upon Tyne, United Kingdom

**Keywords:** neuroblastoma, DNA methylation, prognosis, biomarker

## Abstract

Accurate assessment of neuroblastoma outcome prediction remains challenging. Therefore, this study aims at establishing novel prognostic tumor DNA methylation biomarkers. In total, 396 low- and high-risk primary tumors were analyzed, of which 87 were profiled using methyl-CpG-binding domain (MBD) sequencing for differential methylation analysis between prognostic patient groups. Subsequently, methylation-specific PCR (MSP) assays were developed for 78 top-ranking differentially methylated regions and tested on two independent cohorts of 132 and 177 samples, respectively. Further, a new statistical framework was used to identify a robust set of MSP assays of which the methylation score (i.e. the percentage of methylated assays) allows accurate outcome prediction. Survival analyses were performed on the individual target level, as well as on the combined multimarker signature. As a result of the differential DNA methylation assessment by MBD sequencing, 58 of the 78 MSP assays were designed in regions previously unexplored in neuroblastoma, and 36 are located in non-promoter or non-coding regions. In total, 5 individual MSP assays (located in *CCDC177*, *NXPH1*, *lnc-MRPL3-2, lnc-TREX1-1* and one on a region from chromosome 8 with no further annotation) predict event-free survival and 4 additional assays (located in *SPRED3*, *TNFAIP2*, *NPM2* and *CYYR1*) also predict overall survival. Furthermore, a robust 58-marker methylation signature predicting overall and event-free survival was established. In conclusion, this study encompasses the largest DNA methylation biomarker study in neuroblastoma so far. We identified and independently validated several novel prognostic biomarkers, as well as a prognostic 58-marker methylation signature.

## INTRODUCTION

Neuroblastoma (NB), a childhood tumor that originates from precursor cells of the sympathetic nervous system, is a heterogeneous disease with prognosis ranging from excellent long-term survival to high-risk with fatal outcome. In order to determine the most appropriate treatment modalities for each patient, patients are stratified into risk groups at the time of diagnosis, based on combinations of clinical (age of the patient, stage of the tumor) and biological (*MYCN* amplification status, DNA index, histopathology) parameters [1]. Use of this risk classification system has shown that patients characterized by the same clinicobiological parameters can have different disease outcomes, indicating that accurate assessment of prognosis of NB patients still remains difficult [2-4]. Therefore, additional prognostic markers are warranted, allowing a more accurate risk estimation and more rapid identification of those patients who will not benefit from current treatments.

Molecular alterations of the epigenome, especially DNA methylation, have emerged as alternative targets of biomarker research. DNA methylation biomarkers potentially have great clinical value due to the stable nature of DNA. For this reason, there are many relevant applications of DNA methylation biomarkers in cancer. For example, they could be used for early tumor detection, tumor classification, stratification of treatment, tumor recurrence and patient prognosis, as well as predicting and monitoring a patient's response to treatment (detailed review in reference [5]). In NB, several prognostic single-gene methylation biomarkers have been reported, e.g. promoter methylation of *TNFRSF10D*, *CASP8*, *ZMYND10*, *RASSF1A*, *KRT19*, *GNAS*, *HIST1H3C, RB1* and *TDGF1* [6-11]. Furthermore, a CpG island methylator phenotype (CIMP), described as the aberrant and concordant methylation of multiple promoter CpG islands, has been shown to be of prognostic significance [12-16].

In this study, we aim to assess the primary NB tumor methylome in a genome-wide manner to identify differentially methylated regions (DMRs) between the prognostic patient groups, and to use these DMRs to establish and validate new and valuable biomarkers.

## RESULTS

### Methyl-CpG-binding domain (MBD) sequencing of primary tumors prioritizes differentially methylated regions (DMRs) between patient subgroups

**Figure 1:**
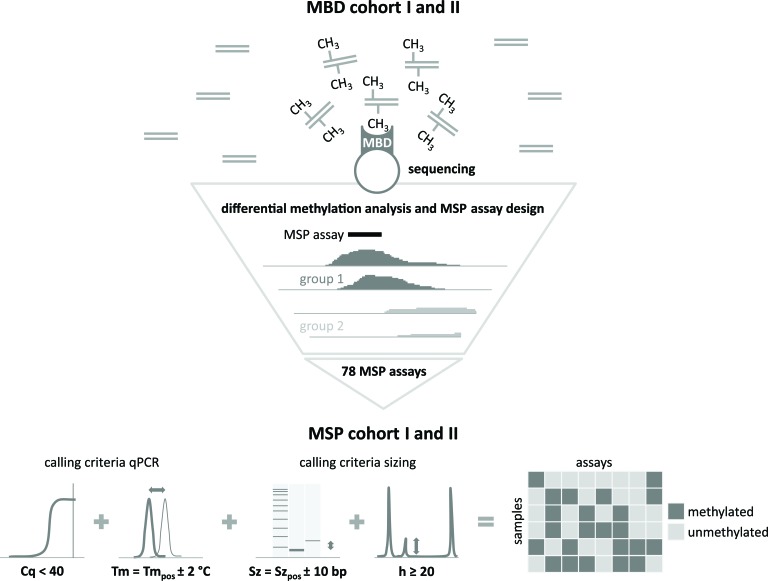
Schematic representation of the study design Differentially methylated regions (DMRs) between the prognostic patient groups are identified by methyl-CpG-binding (MBD) sequencing on MBD cohort I and II. For the top candidate prognostic DMRs, the MBD sequencing data were visualized in order to locate the most informative region for methylation-specific PCR (MSP) assay design. These assays were subsequently tested on MSP cohort I and II. By applying specific methylation calling criteria [10], a binary dataset for each of these cohorts was constructed, which was subsequently used for survival analyses. Cq: quantification cycle, Tm: melting temperature; Sz: size; h: height. The subscript pos refers to the data of the positive control sample.

The study design is schematically represented in Figure 1. In the discovery phase, two independent cohorts of 42 (MBD cohort I) and 45 (MBD cohort II) primary NB tumors, selected for risk classification and survival (low-risk survivors (LR-SURV), high-risk survivors (HR-SURV) and high-risk deceased (HR-DOD)), were analyzed by methyl-CpG-binding domain (MBD) sequencing (Supplemental Table 1A and B). Sheared input DNA was enriched towards methylated fragments using the high affinity of the MBD of the MeCP2 protein towards methylated cytosines. These methylation-enriched fractions, as well as the input (non-MBD-enriched) DNA of MBD cohort II were then further studied by next-generation sequencing. After raw data analyses, differentially methylated regions (DMRs) between patient subgroups were detected using DESeq, which uses count data as input. The following patient subgroups were compared: HR-SURV versus HR-DOD (on the entire cohorts, as well as on the high-risk *MYCN* amplified (HR-MYCN1) and non-amplified (HR-MYCN0) cohorts only), LR-SURV versus HR-DOD, and HR-MYCN0 versus HR-MYCN1 (Supplemental Table 2). The same analyses were performed on the input sample data in order to estimate the background signal and exclude falsely identified DMRs. The DESeq analyses yield for each region of interest the mean normalized counts per patient group, as well as the log2FoldChange and p-value for the statistical significance of the difference. By calculating the π-value (π = −ln pval * log2 fold change [17]) for each of these regions, a new significance score was defined, which was then used to rank the candidate prognostic DMRs. Hierarchical cluster analysis using normalized counts of the top-ranking DMRs yielded two sample clusters which mainly correspond to the patient groups used in the differential methylation analysis, highlighting the capability of our MBD sequencing analysis strategy in identifying biomarker candidates (examples shown in Supplemental Figure 1).

### Methylation-specific PCR (MSP) assays are designed and tested on two independent cohorts

MBD sequencing data of the top-ranking DMRs (promoter regions and 5 kb windows) from the different prognostic comparisons were visualized in the Integrative Genomics Viewer (IGV; [18]) in order to locate the most informative (discriminative) region for MSP primer design (Figure 1). The importance of this step is illustrated by the promoter region of *HNRNPH1,* which was identified as differentially methylated between HR-SURV and HR-DOD patients, and LR-SURV and HR-DOD patients (Supplemental Figure 2). MBD regions for which no clear discriminative region could be identified were excluded from further analyses and only DMRs hypermethylated in HR-DOD or HR-MYCN1 samples were considered for further evaluation. In total, 78 MSP assays (Supplemental Table 2) were designed, analytically validated and tested on 19 NB cell lines (Supplemental Table 3), positive and negative controls (the (*in vitro* methylated) HCT-116 DKO cell line), along with two independent cohorts of 148 (MSP cohort I) and 202 (MSP cohort II) primary NB samples assigned to one of the three defined prognostic patient groups (Supplemental Table 4). Also the *ACTB* primer pair, a control assay that does not contain CpG sites and thus should always generate a PCR product, was tested on these samples to confirm successful DNA preparation (bisulfite treatment and amplification). In total, 16 samples of MSP cohort I and 25 samples of MSP cohort II failed for this assay, probably due to low DNA quality, and were therefore excluded from the study.

### MSP confirms the validity of MBD sequencing in identifying candidate methylation biomarkers

In both MSP cohort I and II, primary tumor samples of HR-DOD and HR-MYCN1 patients show more methylation events compared to either survivors (p = 0.001 for both cohorts; Supplemental Figure 3A and 3B) and HR-MYCN0 patients (p < 0.001 for both cohorts; Supplemental Figure 3C and 3D), respectively. This again confirms the validity of MBD sequencing data in identifying candidate markers, as all MSP assays were designed in regions identified in the MBD sequencing data as being hypermethylated in HR-DOD or HR-MYCN1 patients. To further strengthen MBD sequencing as a powerful technology for identification of genome-wide differential methylation, the genomic locations of the in-house designed MSP assays were compared to the genomic locations of the cytosines interrogated on the Infinium HumanMethylation450 BeadChip Kit (HM450 array; Illumina). Of note, 58 MSP assays (74.36%) do not overlap with an interrogated cytosine on the HM450 array, and would thus not have been identified using this array technology (e.g. promoter region of *UHRF2* in Supplemental Figure 4). Also, 36 MSP assays (46.15%) are located in non-promoter or non-coding regions.

### Survival analyses on the individual MSP assay level identify new prognostic biomarkers

Overall, the percentage of methylated samples per MSP assay ranges from 96.97% to 2.27% in MSP cohort I, and from 97.18% to 1.70% in MSP cohort II, and variable percentages between the prognostic patient groups are detected (Supplemental Table 4). The results of the survival analyses (log-rank test) on each individual MSP assay and the different patient (sub)cohorts are indicated in Supplemental Table 2. Although the survival analyses on the high-risk subgroups did not yield significant results, analyses on the entire cohorts identified 9 individual prognostic MSP assays for overall survival (OS) and 5 assays for event-free survival (EFS) that were significantly detected in both MSP cohort I and II (Table 1). For EFS, these assays are located in the promoter region or gene body of *CCDC177* and *NXPH1*, and the long non-coding RNAs *lnc-MRPL3-2* and *lnc-TREX1-1*. The additional prognostic assays for OS are located in the promoter region of *SPRED3*, *TNFAIP2*, *NPM2* and *CYYR1*. For MSP assay011, which has prognostic value for both OS and EFS, the amplicon is located on chr8:143 498 349 - 143 498 469 (flanking genes are *TSNARE1* (downstream on ± 14 kb) and *BAI1* (upstream on ± 47 kb on opposite strand)). The corresponding results of the univariable logistic regression analyses are also shown in Table 1, and associations between the prognostic DNA methylation biomarkers and established prognostic NB risk factors (*MYCN* amplification, age at diagnosis (both 12 and 18 month cutoff) and International Neuroblastoma Staging System (INSS) stage [19]) are shown in Table 2.

**Table 1:** Survival analyses on the individual MSP assay level identify new biomarkers for overall and event-free survival

		MSP cohort I				MSP cohort II			
overall survival	log-rank	univariable logistic regression			log-rank	univariable logistic regression		
variable	p	p	OR	95% CI	p	p	OR	95% CI
assay006 (*SPRED3*)	0.005	0.043	2.26	1.03 - 4.96	0.014	0.030	2.08	1.08 - 4.04
assay008 (*TNFAIP2*)	0.008	0.009	3.13	1.33 - 7.40	0.025	0.020	2.28	1.14 - 4.57
assay011	0.002	0.001	3.56	1.64 - 7.75	< 0.001	< 0.001	3.82	1.81 - 8.08
assay062 (*NPM2*)	0.021	0.010	4.19	1.41 - 12.46	0.038	0.036	2.38	1.06 - 5.33
assay087 (*NXPH1*)	0.014	0.043	2.26	1.03 - 4.96	0.003	0.004	2.60	1.37 - 4.95
assay108 (*CYYR1*)	0.024	0.004	3.46	1.48 - 8.13	0.046	0.040	1.93	1.03 - 3.61
assay111 (*CCDC177*)	0.002	< 0.001	3.68	1.71 - 7.89	0.020	0.022	2.11	1.11 - 3.99
assay113 (*lnc-MRPL3-2*)	0.002	0.004	3.03	1.43 - 6.43	0.034	0.056	1.84	0.98 - 3.45
assay116 (*lnc-TREX1-1*)	0.008	0.056	3.27	0.97 - 10.98	0.004	0.021	2.87	1.18 - 7.01
**event-free survival**	**log-rank**	**univariable logistic regression**			**log-rank**	**univariable logistic regression**		
**variable**	**p**	**p**	**OR**	**95% CI**	**p**	**p**	**OR**	**95% CI**
assay011	0.007	0.006	2.77	1.35 - 5.70	< 0.001	< 0.001	4.18	2.02 - 8.67
assay087 (*NXPH1*)	0.018	0.017	2.58	1.19 - 5.62	0.003	0.004	2.56	1.35 - 4.83
assay111 (*CCDC177*)	< 0.001	< 0.001	4.94	2.33 - 10.48	0.007	0.004	2.52	1.34 - 4.74
assay113 (*lnc-MRPL3-2*)	0.035	0.040	2.12	1.04 - 4.32	0.038	0.030	1.98	1.07 - 3.67
assay116 (*lnc-TREX1-1*)	0.022	0.060	3.33	0.95 - 11.70	0.019	0.055	2.38	0.98 - 5.80

Note. For each individual MSP assay, the log-rank p-values, and the p-value, odds ratio (OR) and 95% confidence interval (CI) of the univariable logistic regression analyses are shown. Methylation of the individual markers is associated with worse overall and event-free survival.

**Table 2:** The nine individual prognostic MSP assays are differentially methylated between patient groups with distinct neuroblastoma risk factors

MSP cohort I										
factor - number (percentage)		assay006 (*SPRED3*)	assay008 (*TNFAIP2*)	assay011	assay062 (*NPM2*)	assay087 (*NXPH1*)	assay108 (*CYYR1*)	assay111 (*CCDC177*)	assay113 (*lnc-MRPL3-2*)	assay116 (*lnc-TREX1-1*)
INSS stage	stage 1 (n = 27)	3 (11.11)	0 (0.00)	6 (22.22)	0 (0.00)	2 (7.41)	1 (3.70)	2 (7.41)	8 (29.63)	1 (3.70)
	stage 2 (n = 18)	1 (5.56)	1 (5.56)	7 (38.89)	1 (5.56)	1 (5.56)	0 (0.00)	5 (27.78)	4 (22.22)	0 (0.00)
	stage 3 (n = 33)	15 (45.45)	10 (30.30)	17 (51.52)	5 (15.15)	16 (48.48)	11 (33.33)	16 (48.48)	13 (39.39)	4 (12.12)
	stage 4 (n = 54)	18 (33.33)	17 (31.48)	35 (64.81)	10 (18.52)	18 (33.33)	17 (31.48)	32 (59.26)	28 (51.85)	7 (12.96)
*MYCN* amplification status	MYCN0 (n = 96)	11 (11.46)	11 (11.46)	39 (40.63)	5 (5.21)	12 (12.50)	13 (13.54)	29 (30.21)	28 (29.17)	2 (2.08)
	MYCN1 (n = 36)	26 (72.22)	17 (47.22)	26 (72.22)	11 (30.56)	25 (69.44)	16 (44.44)	26 (72.22)	25 (69.44)	10 (27.78)
age at diagnosis	≤ 12 months (n = 54)	6 (11.11)	1 (1.85)	17 (31.48)	1 (1.85)	9 (16.67)	1 (1.85)	11 (20.37)	11 (20.37)	1 (1.85)
	> 12 months (n = 78)	31 (39.74)	27 (34.62)	48 (61.54)	15 (19.23)	28 (35.90)	28 (35.90)	44 (56.41)	42 (53.85)	11 (14.10)
	≤ 18 months (n = 63)	10 (15.87)	5 (7.94)	22 (34.92)	1 (1.59)	10 (15.87)	2 (3.17)	14 (22.22)	16 (25.40)	1 (1.59)
	> 18 months (n = 39)	27 (39.13)	23 (33.33)	43 (62.32)	15 (21.74)	27 (39.13)	27 (39.13)	41 (59.42)	37 (53.62)	11 (15.94)
**factor - statistics (p)**		**assay006 (*SPRED3*)**	**assay008 (*TNFAIP2*)**	**assay011**	**assay062 (*NPM2*)**	**assay087 (*NXPH1*)**	**assay108 (*CYYR1*)**	**assay111 (*CCDC177*)**	**assay113 (*lnc-MRPL3-2*)**	**assay116 (*lnc-TREX1-1*)**
INSS stage		0.002	< 0.001	0.003	0.056	< 0.001	< 0.001	< 0.001	0.085	0.302
*MYCN* amplification status		< 0.001	< 0.001	0.002	< 0.001	< 0.001	< 0.001	< 0.001	< 0.001	< 0.001
age at diagnosis (cutoff 12 months)		< 0.001	< 0.001	0.001	0.002	0.018	< 0.001	< 0.001	< 0.001	0.027
age at diagnosis (cutoff 18 months)		0.004	< 0.001	0.002	< 0.001	0.004	< 0.001	< 0.001	0.001	0.005
**MSP cohort II**										
**factor - number (percentage)**		**assay006 (*SPRED3*)**	**assay008 (*TNFAIP2*)**	**assay011**	**assay062 (*NPM2*)**	**assay087 (*NXPH1*)**	**assay108 (*CYYR1*)**	**assay111 (*CCDC177*)**	**assay113 (*lnc-MRPL3-2*)**	**assay116 (*lnc-TREX1-1*)**
INSS stage	stage 1 (n = 27)	2 (7.41)	1 (3.70)	7 (25.93)	0 (0.00)	3 (11.11)	10 (37.04)	11 (40.74)	10 (37.04)	1 (3.70)
	stage 2 (n = 17)	4 (23.53)	3 (17.65)	8 (47.06)	0 (0.00)	4 (23.53)	5 (29.41)	6 (35.29)	4 (23.53)	0 (0.00)
	stage 3 (n = 27)	7 (25.93)	6 (22.22)	14 (51.85)	5 (18.52)	9 (33.33)	12 (44.44)	9 (33.33)	14 (51.85)	6 (22.22)
	stage 4 (n = 103)	41 (39.81)	35 (33.98)	83 (80.58)	24 (23.30)	48 (46.60)	50 (48.54)	66 (64.08)	56 (54.37)	16 (15.53)
*MYCN* amplification status	MYCN0 (n = 115)	11 (9.57)	14 (12.17)	63 (54.78)	11 (9.57)	22 (19.13)	39 (33.91)	47 (40.87)	43 (37.39)	7 (6.09)
	MYCN1 (n = 60)	43 (71.67)	30 (50.00)	48 (80.00)	18 (30.00)	40 (66.67)	37 (61.67)	43 (71.67)	40 (66.67)	16 (26.67)
age at diagnosis	≤ 12 months (n = 53)	1 (1.89)	1 (1.89)	14 (26.42)	0 (0.00)	8 (15.09)	12 (22.64)	10 (18.87)	11 (20.75)	3 (5.66)
	> 12 months (n = 124)	53 (42.74)	44 (35.48)	99 (79.84)	29 (23.39)	56 (45.16)	65 (52.42)	82 (66.13)	73 (58.87)	20 (16.13)
	≤ 18 months (n = 74)	10 (13.51)	4 (5.41)	27 (36.49)	2 (2.70)	14 (18.92)	23 (31.08)	22 (29.73)	22 (29.73)	8 (10.81)
	> 18 months (n = 103)	44 (42.72)	41 (39.81)	86 (83.50)	27 (26.21)	50 (48.54)	54 (52.43)	70 (67.96)	62 (60.19)	15 (14.56)
**factor - statistics (p)**		**assay006 (*SPRED3*)**	**assay008 (*TNFAIP2*)**	**assay011**	**assay062 (*NPM2*)**	**assay087 (*NXPH1*)**	**assay108 (*CYYR1*)**	**assay111 (*CCDC177*)**	**assay113 (*lnc-MRPL3-2*)**	**assay116 (*lnc-TREX1-1*)**
INSS stage		0.006	0.005	< 0.001	0.002	0.003	0.439	0.004	0.066	0.066
*MYCN* amplification status		< 0.001	< 0.001	0.001	0.001	< 0.001	0.001	< 0.001	< 0.001	< 0.001
age at diagnosis (cutoff 12 months)		< 0.001	< 0.001	< 0.001	< 0.001	< 0.001	< 0.001	< 0.001	< 0.001	0.085
age at diagnosis (cutoff 18 months)		< 0.001	< 0.001	< 0.001	< 0.001	< 0.001	0.006	< 0.001	< 0.001	0.505

Note. For each of the nine individual prognostic MSP assays the number (percentage) of methylated samples in each stratum of MSP cohort I and II is given. P-values are according to the Fisher's exact test. INSS: International Neuroblastoma Staging System; MYCN0: *MYCN* non-amplified; MYCN1: *MYCN*amplified.

### A 58-marker methylation signature with accompanying methylation score cutoff of 25% predicts overall and event-free survival

**Figure 2:**
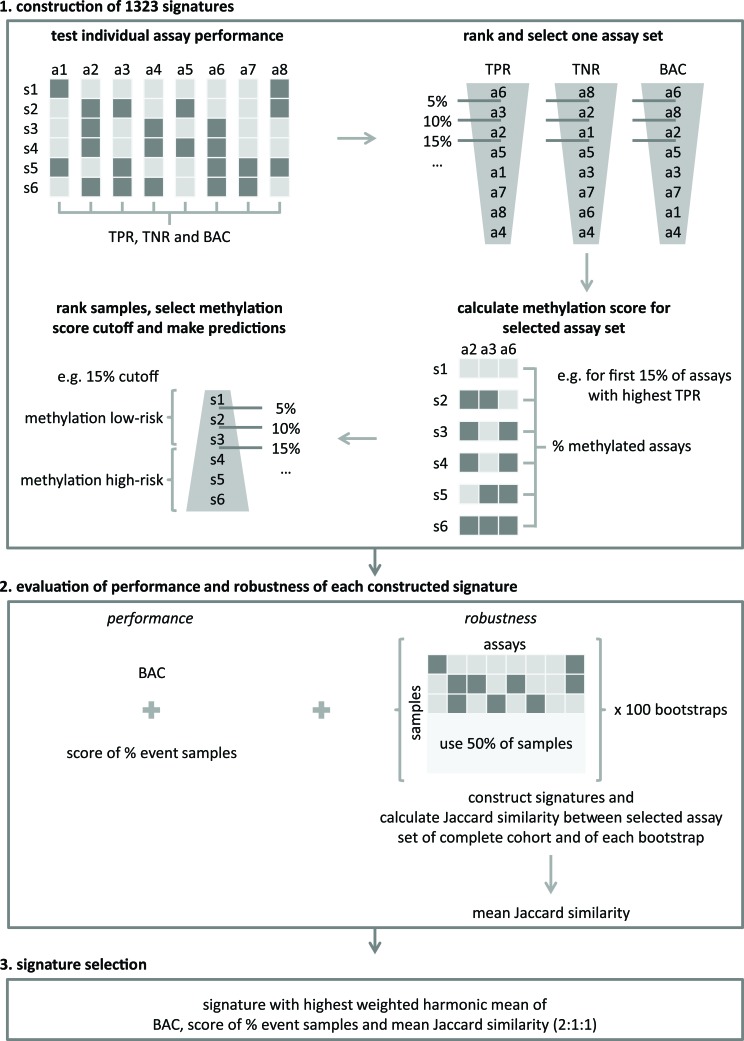
A new statistical framework was developed to identify a robust multimarker signature for accurate outcome prediction The framework consists of three major steps: (1) signatures construction, (2) evaluation of the performance and robustness of the constructed signatures and (3) the selection of the final signature. Details of every step are described in the Materials and Methods section. a: assay; s: sample; TPR: true positive rate (sensitivity); TNR: true negative rate (specificity); balanced accuracy (BAC).

**Figure 3:**
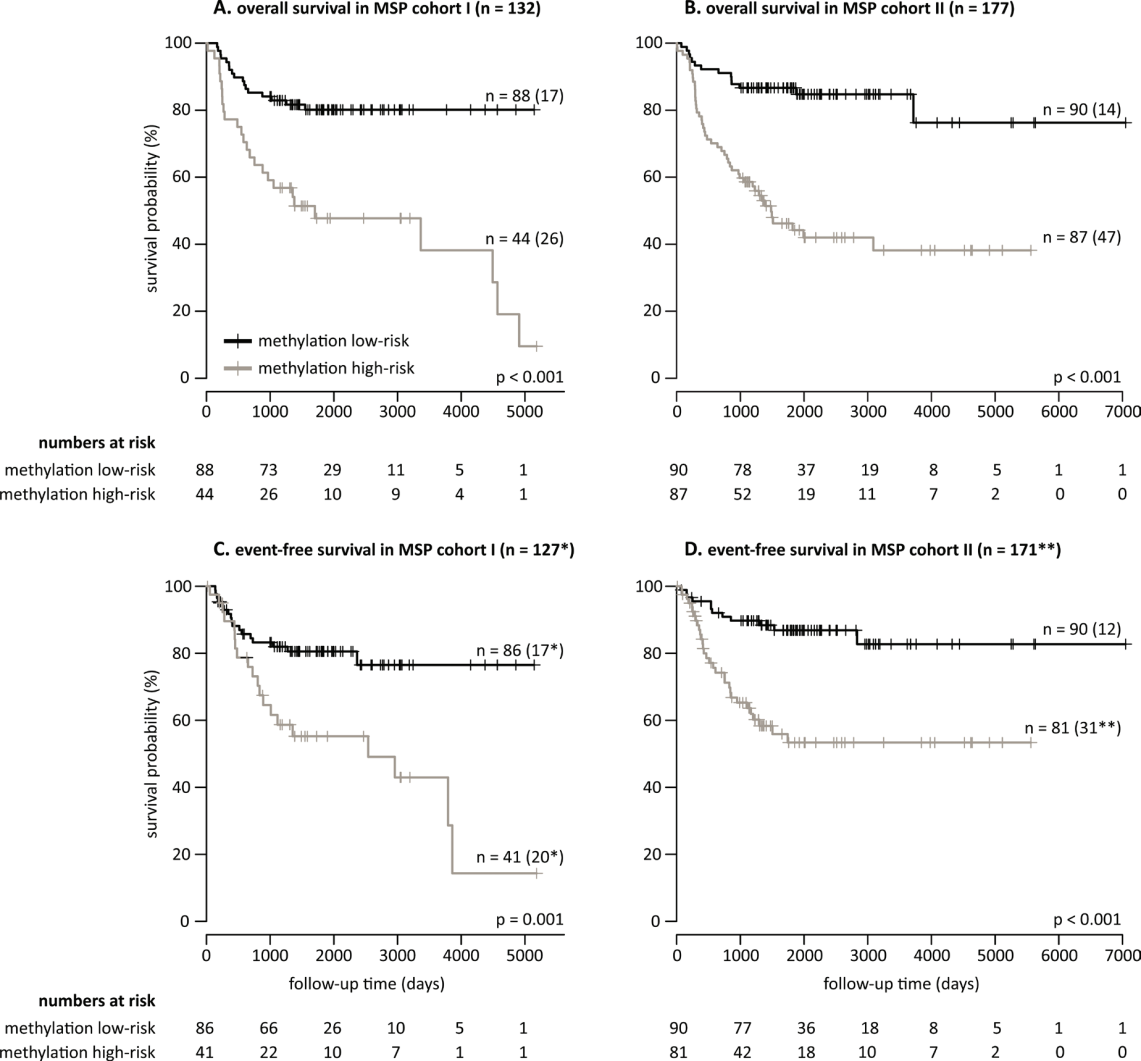
A robust 58-marker methylation signature and methylation score of 25% predicts overall and event-free survival Kaplan-Meier curves and log-rank p-values for overall survival (MSP cohort I in A. and MSP cohort II in B.) and event-free survival (MSP cohort I in C. and MSP cohort II in D.) are shown. The numbers of patients at methylation low- and high-risk as predicted by the 58-marker signature are indicated. The numbers in parentheses in the plots refer to the number of patients that experienced an event (death of disease for overall survival, and relapse, progression or death of disease for event-free survival). *Missing follow-up time for two methylation low-risk patients and three methylation high-risk patients. **Missing follow-up time for five methylation high-risk patients, and event status and follow-up time for one patient.

As all MSP assays were designed in regions identified as hypermethylated in HR-DOD or HR-MYCN1 samples and as the MSP data show association with outcome (Supplemental Figure 3A-3D), the possibility of establishing a robust and accurate multimarker signature for OS and EFS based on the number of methylation events was explored. To this purpose, a new statistical framework was developed, which allows identification of a robust set of MSP assays of which the methylation scores (i.e. the percentage of methylated assays in each sample) allow accurate outcome prediction (details in Materials and Methods and Figure 2). The signature was trained on MSP cohort I and tested on MSP cohort II. For the high-risk subgroups, the resulting signature was not prognostic, but using the entire sample cohorts, a set of 58 MSP assays (Supplemental Table 4) with a methylation score cutoff of 25% was put forward and shown to significantly predict OS (p < 0.001 for both cohorts, log-rank test) and EFS (p = 0.001 for MSP cohort I and p < 0.001 for MSP cohort II). For MSP cohort I, OS at 5 years of follow-up is 80.14% (95% confidence interval (CI) 72.06 - 89.11) for the group of patients at methylation low-risk, compared to 47.74% (34.43 - 66.18) for the group of patients at methylation high-risk. The 5-year EFS is 80.54% (72.40 - 89.61) and 55.22% (40.92 - 74.51) in the methylation low- and high-risk groups, respectively. For MSP cohort II, OS at 5 years of follow-up is 86.67% (79.92 - 93.98) for the methylation low-risk group, compared to 44.20% (34.14 - 57.23) for the methylation high-risk group. Here, the 5-year EFS is 86.86% (79.86 - 94.47) and 53.34% (42.06 - 67.65) in the methylation low- and high-risk groups, respectively. The corresponding Kaplan-Meier curves are depicted in Figure 3. Power analyses using these survival rates illustrate that the MSP cohorts contain sufficient numbers of samples to obtain 90% power at 5% significance level. The signature has a balanced accuracy (BAC) of 70.12% for OS and 65.71% for EFS on MSP cohort I. On MSP cohort II, these values are 71.28% and 67.97%, respectively. Univariable logistic regression analyses also illustrate that the signature predicts OS and EFS, and multivariable logistic regression analyses show that the signature is a significantly independent predictor of OS in MSP cohort II after controlling for known risk factors (Supplemental Table 5). Associations between the signature predictions and established NB risk factors are shown in Table 3.

**Table 3:** The 58-marker signature predictions are associated with established neuroblastoma risk factors

MSP cohort I			
factor - number (percentage)		OS signature prediction	EFS signature prediction
INSS stage	stage 1 (n = 27)	0 (0.00)	0 (0.00)
	stage 2 (n = 18)	1 (5.56)	1 (5.56)
	stage 3 (n = 33)	15 (45.45)	15 (45.45)
	stage 4 (n = 54)	28 (51.85)	28 (51.85)
*MYCN* amplification status	MYCN0 (n = 96)	17 (17.71)	17 (17.71)
	MYCN1 (n = 36)	27 (75.00)	27 (75.00)
age at diagnosis	≤ 12 months (n = 54)	5 (9.26)	5 (9.26)
	> 12 months (n = 78)	39 (50.00)	39 (50.00)
	≤ 18 months (n = 63)	8 (12.70)	8 (12.70)
	> 18 months (n=69)	36 (52.17)	36 (52.17)
**MSP cohort II**			
**factor - number (percentage)**		**OS signature prediction**	**EFS signature prediction**
INSS stage	stage 1 (n = 27)	4 (14.81)	4 (14.81)
	stage 2 (n = 17)	4 (23.53)	4 (23.53)
	stage 3 (n = 27)	7 (25.93)	7 (25.93)
	stage 4 (n = 103*)	72 (69.90)	71 (69.61)
*MYCN* amplification status	MYCN0 (n = 115)	38 (33.04)	38 (33.04)
	MYCN1 (n = 60*)	47 (78.33)	46 (77.97)
age at diagnosis	≤ 12 months (n = 53)	1 (1.89)	1 (1.89)
	> 12 months (n = 124*)	86 (69.35)	85 (69.11)
	≤ 18 months (n = 74)	9 (12.16)	9 (12.16)
	> 18 months (n = 103*)	78 (75.73)	77 (75.49)

Note. For both OS and EFS, the number (percentages) of methylation high-risk samples in each stratum of MSP cohort I and II is given. All associations are statistically significant (p < 0.001; Fisher's exact test). OS: overall survival; EFS: event-free survival; INSS: International Neuroblastoma Staging System; MYCN0: *MYCN* non-amplified; MYCN1: *MYCN* amplified. *Missing event-free survival status for one patient.

## DISCUSSION

*MYCN* amplification was identified as first genetic prognostic marker, in addition to age at diagnosis and tumor stage, which is still used today in therapeutic stratification [1]. Further studies have attempted to explore additional parameters to improve prognostic classification. Most notably, these include large chromosomal imbalances as well as transcriptome-based gene signatures. Given the low mutation burden, more recent sequencing efforts did not deliver significant novel tools for prognostic stratification [20], although *ALK* mutation status is of importance for including patients for targeted therapy with ALK inhibitors. Recent studies have shown that NB biology is also strongly determined by the epigenetic profile of the tumor, which has paved the way for prognostic DNA methylation biomarker research. During the past years, multiple prognostic single-gene methylation biomarkers have been described in NB; also a so-called CpG island methylator phenotype (CIMP) was found to be of prognostic value [6-16]. Here, we studied the NB methylome in a genome-wide manner to establish and validate novel prognostic biomarkers for OS and EFS.

Several features contribute to the novel and comprehensive aspect of our study. A first important feature is the number of analyzed tumor samples. In total, 396 primary tumors were included, which is the largest series studied to date. Most reported studies only rely on NB cell lines or on a relatively limited number of tumors in the discovery phase and thus fall short in covering the NB heterogeneity, or lack independent validation on large sample cohorts. Of note, previous studies on mRNAs and microRNAs in NB have emphasized that biomarkers are of little or no utility if they are not validated on an independent patient cohort [21,22]. Here, MBD sequencing was applied to 87 primary tumors, carefully selected for risk classification, allowing optimal biomarker discovery, and two independent cohorts of 132 and 177 primary tumors were used to test the selected candidate biomarkers. Power analyses further emphasize that these large sample collections result in adequate power of the study.

Another important feature is that we made use of MBD sequencing of primary NB tumors in the discovery phase to identify novel biomarker candidates. Compared to the Illumina methylation arrays, which were previously applied to NB tumors, MBD sequencing interrogates more CpGs (approximately 18% of all CpGs versus < 2% for the arrays [23]) and thus allows extension of the biomarker discovery phase to previously unexplored regions. MBD sequencing also has higher genomic coverage than methodologies based on antibodies (methylated DNA immunoprecipitation (MeDIP)) [24]. This genome-wide assessment of the DNA methylation pattern is reflected in the final selection of MSP assays, as we have shown that most of the assays would not have been identified using the HM450 array and that a substantial part of the assays is located in non-promoter or non-coding regions. These findings support previous studies in other cancer types that show that it is important to extend the search for potentially clinical applicable DNA methylation biomarker to the entire methylome rather than focussing on promoter CpG islands of which methylation is in most cases inversely correlated to their transcriptional activity [5].

The prognostic relevance of the selected candidate biomarkers was further analyzed in two large independent cohorts using our previously established high-throughput and semi-automated MSP pipeline [10]. As these cohorts include a considerable number of both high-risk survivors and non-survivors, the candidates could not only be tested on the entire sample cohorts, but also on the high-risk cohorts only. This analysis is very valuable, for the reason that the need for prognostic biomarkers is the highest within this group of patients. However, although differential methylation analyses and hierarchical clustering on the MBD sequencing data illustrate that high-risk survivors and high-risk non-survivors show different methylation patterns, the MSP screens did not identify markers that were significantly prognostic in both MSP high-risk cohort I and II. Importantly, this does not mean that high-risk DNA methylation biomarkers cannot be found. It only indicates that the methylation differences in the DMRs (of 2 kb or 5 kb) in the MBD sequencing data between these high-risk groups are too subtle to be easily translated in an MSP assay which only interrogates a few CpGs. Therefore, the possibility of establishing high-risk methylation biomarkers based on genome-wide bisulfite sequencing, which allows analysis of the methylome at the single CpG level, should be addressed in the future. These future studies might also benefit from focussing on more homogeneous high-risk patient groups, for example by only studying *MYCN* amplified or non-amplified samples, as the heterogeneity within our high-risk cohort might also have counteracted the possibility of establishing high-risk DNA methylation biomarkers.

Nevertheless, our validation efforts allowed robust identification of prognostic assays on the entire patient cohorts. Newly discovered individual prognostic methylation biomarkers for event-free survival (EFS) are *CCDC177* and *NXPH1*, and *SPRED3*, *TNFAIP2*, *NPM2* and *CYYR1* for overall survival (OS). Interestingly, some of these biomarkers are linked with neuronal processes and/or have already been described in other tumor types. For example, *NXPH1* encodes the neurexophilin 1 protein that forms a very tight complex with alpha neurexins, a group of proteins that promote adhesion between dendrites and axons, and methylation of this gene was previously described as potential diagnostic biomarker for breast cancer [26]. *TNFAIP2* (tumor necrosis factor, alpha-induced protein 2) was also found to be hypermethylated in colorectal cancer [27] and *NPM2* (nucleophosmin/nucleoplasmin 2) in melanoma [28] and acute myeloid leukemia [29]. Alterations of sequence and expression of *CYYR1* (cysteine/tyrosine-rich 1) were previously observed in neuroendocrine tumors [30]. Remarkably, also three non-coding methylation biomarkers for OS and EFS were identified (*lnc-MRPL3-2*, *lnc-TREX1-1* and assay011). Assay011 is located on chr8:143 498 349 - 143 498 469, but further annotation is not available for this region. These findings again underscore the importance of screening the entire methylome for biomarker discovery. Of note, the role of methylation of these non-promoter CpGs in NB is currently unclear and should also be topic of further investigation, as it has been shown that DNA methylation outside promoters may also be crucial for gene regulation [25]. Clearly, this might reveal new aspects of NB tumorigenesis.

Finally, a new statistical framework was applied to identify a robust set of MSP assays of which the methylation scores of the samples allow accurate outcome prediction. Both for OS and EFS, a 58-marker signature with a methylation score cutoff of 25% was selected based on the data of MSP cohort I. Survival analyses on both MSP cohort I and II indicate that the signature displays prognostic value for OS and EFS, and is a significant independent predictor of OS in MSP cohort II after controlling for established NB risk factors. All newly discovered individual prognostic methylation biomarkers are part of the signature and further inspection of the other assays included in the signature shows biomarkers previously described in other tumor types, as well as genes previously linked to NB, such as *NAV2*, which functions in axonal elongation and is required for all-trans retinoic acid to induce neurite outgrowth in human NB cells [31]. Also in this regard, the present study is unique, since combining multiple individual methylation assays into a single biomarker signature is not previously reported in NB, with the exception of testing the CpG island methylator phenotype (CIMP), but this assay panel was simply adopted from the colorectal cancer research field. Yet, it should be tested whether these established DNA methylation biomarkers can further improve the performance of our 58-marker signature.

In conclusion, the applications of DNA methylation biomarkers in cancer management are versatile and these should definitely be further explored in the context of NB. During the past decades, many efforts have been made to identify prognostic DNA methylation biomarkers for NB, but currently no such biomarkers have made it to the clinic, as they lack comprehensive validation. In our study, we performed genome-wide methylation profiling of primary NB tumors using MBD sequencing to discover novel prognostic methylation biomarkers and subsequently tested top candidates in two independent cohorts using MSP. As such, we comprised 396 patients in total, which greatly increases the validity of the study and makes it the largest DNA methylation biomarker study in NB to date. We robustly identified several novel individual biomarkers for OS and EFS, and could develop a prognostic 58-marker signature of which a methylation score cutoff of 25% allows accurate outcome prediction in the total patient cohorts. Furthermore, on the validation cohort, this signature was an independent predictor of OS after controlling for known NB risk factors, clearly indicating its clinical relevance. As such, this study forms a solid basis for further investigation of our biomarkers and signature in NB subgroups which could not be robustly examined in our cohorts (low-risk non-survivors and more homogeneous high-risk subgroups). Ideally, also the integration with other DNA methylation biomarkers and -omic data should be further explored to fully optimize the assessment of NB prognosis and appropriate stratification of patient treatment.

## MATERIALS AND METHODS

### Neuroblastoma cell lines and primary tumors

In total, 437 primary NB tumor samples were used to establish four independent sample cohorts: MBD cohort I (n = 42), MBD cohort II (n = 45), MSP cohort I (n = 148) and MSP cohort II (n = 202). Also 19 NB cell lines (Supplemental Table 3) were included in the study. All primary tumor samples were assigned to one of three previously defined [10] risk groups based on NB risk parameters (INSS stage, *MYCN* amplification status and age of the patient at diagnosis) and disease outcome: (1) high-risk patients that died of disease (HR-DOD), (2) high-risk survivors (HR-SURV), or (3) low-risk survivors (LR-SURV). Samples were collected at the Centre Léon Bérard (n = 125, Lyon, France), the Hospital Clínico Universitario (n = 86; Valencia, Spain), the Ghent University Hospital (n = 80; Ghent, Belgium), the Sydney Children's Hospital (n = 48; Sydney, Australia), the Institut Curie (n = 37, Paris, France), the Children's Cancer and Leukemia Group (n = 29, Leicester, UK), the Our Lady's Children's Hospital Dublin (n = 13; Dublin, Ireland), the University Hospital Brno (n = 11, Brno, Czech Republic) and the University Children's Hospital Essen (n = 8; Essen, Germany). Detailed clinical characteristics of the patients and a summary across the different subcohorts are given in Supplemental Table 1. The study was approved by the ethical committee of the Ghent University Hospital (approval number: B67020109912).

### Methyl-CpG-binding domain sequencing

DNA fragmentation and MBD-based capturing of 42 (MBD cohort I) and 45 (MBD cohort II) samples were performed as described in [32] and Decock et al., in preparation. Briefly, 200-500 ng sheared DNA was used to enrich for methylated fragments using the MethylCap kit (MBD from MeCP2; Diagenode). For each captured fraction of the samples of MBD cohort I, DNA library preparation was performed using the NEBNext DNA Library Prep Master Mix Set for Illumina (New England Biolabs) in combination with the Multiplexing Sample Preparation Oligonucleotide Kit (Illumina) for paired-end adapter ligation. For the input and enriched fractions of the samples of MBD cohort II, library preparation was automated on an Apollo 324 Next Generation Sequencing Library Preparation System (IntegenX), making use of the PrepX ILM DNA Library Kit (IntegenX) in combination with the Multiplexing Sample Preparation Oligonucleotide Kit. Paired-end sequencing was performed on a Illumina GAIIx (MBD cohort I; PE 2 × 45 bp) and HiSeq2000 (MBD cohort II; PE 2 × 51 bp).

### Methylation-specific PCR

Experimental MSP conditions and methylation calling were done as previously described [10] and are shown in Figure 1. Here, 78 technically validated MSP primer pairs (and the methylation-independent *ACTB* control assay; Supplemental Table 2) were tested on amplified bisulfite-treated DNA from 19 NB cell lines and two independent cohorts of 148 (MSP cohort I) and 202 (MSP cohort II) patients, selected from the previously defined prognostic patient groups.

### Bioinformatics and statistical analyses

#### Methyl-CpG-binding domain sequencing

Raw MBD sequencing data were demultiplexed and converted to FASTQ files. Quality control was performed by FastQC, followed by paired-end read mapping to the human reference genome (hg19) using Bowtie2 [33] and SAMtools [34]. PCR duplicates were marked by Picard and mapping quality control was done by SAMStat [35] and bamUtil. Peaks were called using MACS [36]. Data have been deposited into the Gene Expression Omnibus (GEO; GSE69224 and GSE69243). Count matrices for differential methylation analyses between the prognostic patient subgroups in DESeq [37] were constructed using the R ShortRead [38] and rtracklayer [39] packages. Here, for both MBD cohorts, two count datasets were constructed. The first one represents a table that reports for each MBD-enriched sample the number of mapped reads that are assigned to the promoter region (−1500 bp to +500 bp around transcription start site (TSS)) of the different Ensembl Transcripts (release 68), and the second one to 5 kb genomic windows (2.5 kb overlapping moving windows). Hierarchical clustering was performed using the R gplots and RColorBrewer packages.

#### Methylation-specific PCR

For survival analyses on the MSP data, the Kaplan-Meier method was used to estimate overall and event-free survival (OS and EFS) probabilities, and survival functions were compared with the log-rank test (R survival package). OS time was defined as the time between diagnosis and disease-related death or last follow-up. EFS time is the time between diagnosis and first occurrence of relapse, progression or death of disease, or last follow-up. P-values < 0.05 were considered statistically significant. All individual assays were tested, as well as a multimarker signature that was established on MSP cohort I using a new statistical framework (Figure 2 and Cannoodt et al., in preparation). This framework involves three major steps: (1) the construction of signatures, (2) the evaluation of the performance and robustness of each constructed signature, and (3) the signature selection. The construction of signatures (step 1) is based on the performance of the individual assays, which is evaluated by determining the following statistical metrics: sensitivity (true positive rate (TPR)), specificity (true negative rate (TNR)) and balanced accuracy (BAC). Each of these metrics was subsequently used to rank the assays (from highest to lowest value) and a cutoff, defined by percentiles of the ranked list (from 0% to 100% with 5% increment; 21 possible cutoffs), was applied to select a specific assay set. Then, the methylation score (i.e. the percentage of methylated assays) for each of the samples is calculated and used to rank the samples (from lowest to highest value). Again, a percentage cutoff is applied on the ranked list, which allows making risk predictions for each sample. Samples with a methylation score above the cutoff have a high risk. Samples with a methylation score below the cutoff have a low risk. Given the number of tested metrics to evaluate the individual assay performance (3 possibilities), the number of possible cutoffs to select a specific assay set (21 possibilities), and the number of possible methylation score cutoffs (21 possibilities), 1323 signatures were constructed and further evaluated on their performance and robustness (step 2). The performance of the constructed signatures was examined by determining the BAC, as well as a score that reflects how well the percentage of predicted samples with an event equals the true percentage of samples with an event (score of % event samples in Figure 2). The robustness of the constructed signatures was tested by performing 100 bootstraps, creating a subcohort containing half of the samples. For each of these 100 subcohorts, signatures were constructed as described above and for each combination of parameters the Jaccard similarity index [40] between the selected assay set on the entire cohort and the bootstrap cohort was computed. The robustness of the signature is then reflected in the mean Jaccard similarity of the 100 bootstraps (R caret package). In order to select a final signature (step 3), the performance and robustness metrics are combined in a weighted harmonic mean, and the signature with the highest value is retained. Also power analyses (SAS Power and Sample Size), and univariable and multivariable logistic regression analyses (R survival package) were performed. Included factors in the multivariable analyses are: the *MYCN* amplification status (*MYCN* amplified versus non-amplified as reference), age of the patient at diagnosis (> 18 months versus ≤ 18 months as reference [41]), INSS stage (stage 4 versus not stage 4 as reference) and the signature prediction (methylation high-risk versus methylation low-risk as reference). Associations between the prognostic DNA methylation biomarkers and established NB risk factors (*MYCN* amplification, age at diagnosis (cutoff of 12 months and 18 months) and INSS stage) were determined using Fisher's exact test.

## SUPPLEMENTARY FIGURES AND TABLES












