# Supplementary material for: Methyl-CpG-binding domain sequencing reveals a prognostic methylation signature in neuroblastoma
Source: Oncotarget. 2015 Dec 6;7(2):1960–72. doi: 10.18632/oncotarget.6477 (PMC4811509; doi:10.18632/oncotarget.6477)
Supplement: Supplementary file 1 [file oncotarget-07-1960-s001.pdf]

Methyl-CpG-binding domain sequencing reveals a prognostic methylation signature in neuroblastoma

Supplementary Material

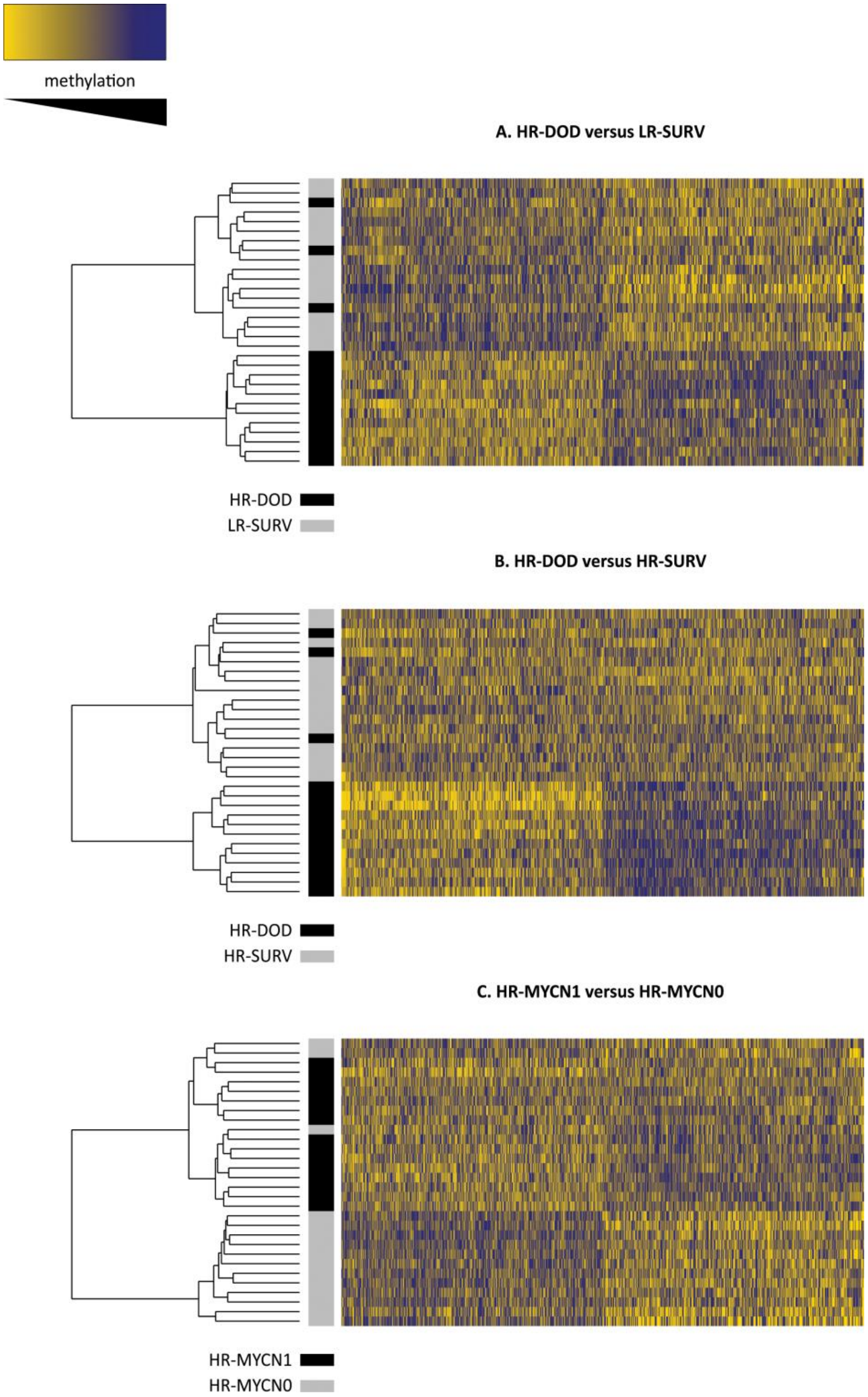

Supplemental figure 1. Hierarchical cluster analyses highlight the capability of the methyl-CpG-binding domain (MBD) sequencing analysis strategy in identifying candidate biomarkers. A. Clustering using the top 500 hyper- and hypomethylated promoter regions in high-risk non-survivors (HR-DOD) compared to low-risk survivors (LR-SURV) in MBD cohort II. **B.** Clustering using the top 500 hyper- and hypomethylated promoter regions in high-risk non-survivors compared to high-risk survivors (HR-SURV) in MBD cohort II. **C.** Clustering using the top 500 hyper- and hypomethylated promoter regions in high-risk *MYCN* amplified (HR-MYCN1) samples compared to high-risk *MYCN* non-amplified samples (HR-MYCN0).

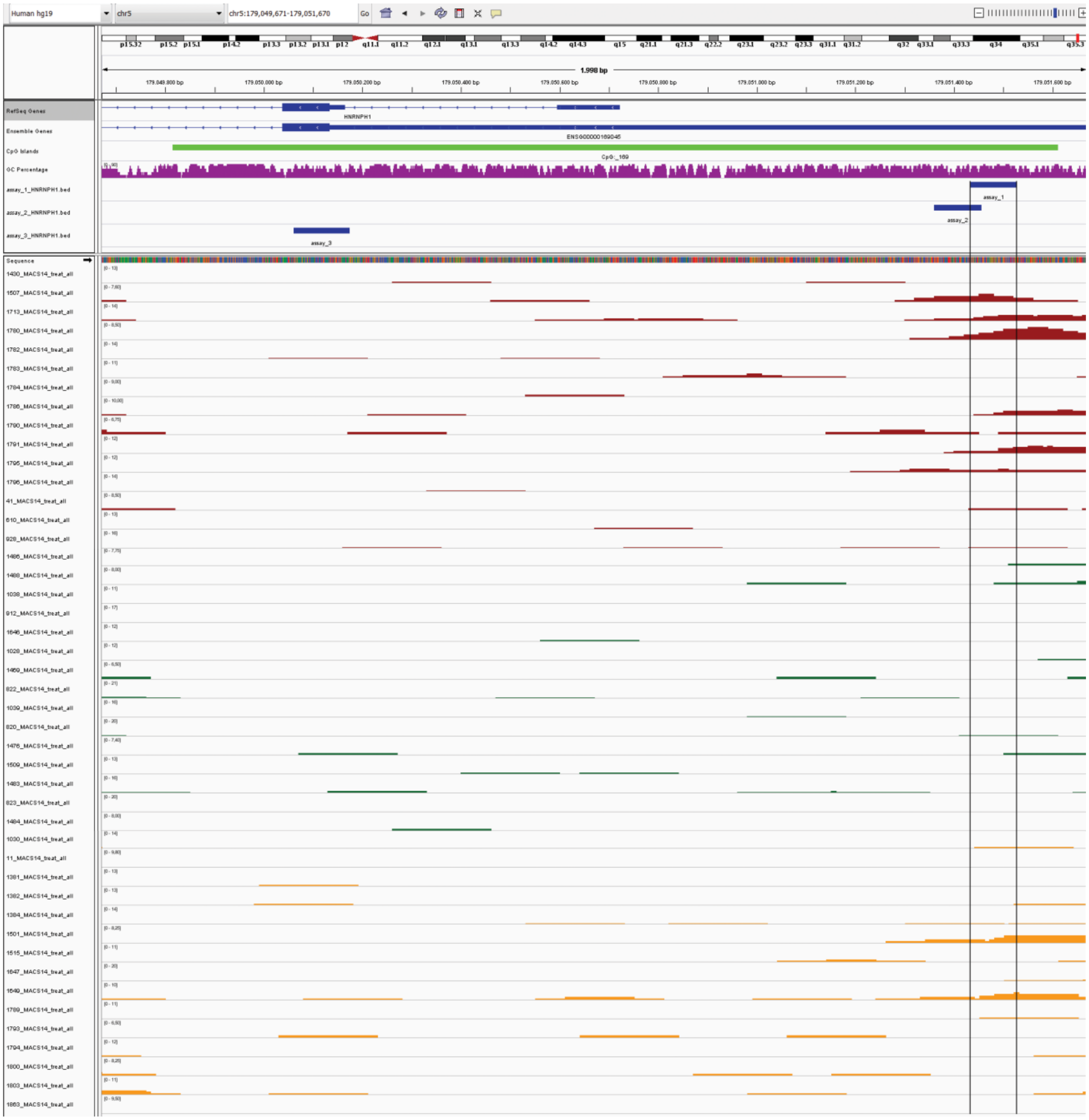

Supplemental figure 2. Visualization of the MBD sequencing data of the *HNRNPH1* promoter region allows identification of the most informative (discriminative) region for MSP assay design. The location of three different MSP assays in the *HNRNPH1* promoter region is shown (blue bars in the upper panel), as well as the number of sequencing tags at each position for each primary tumor of MBD cohort II (lower panel). Assay 1 is located in the region that is most discriminative between high-risk non-

survivors (red) and high-risk survivors (orange)/low-risk survivors (green), while assay 2 and 3 are located in less informative regions (with fuzzy methylation patterns).

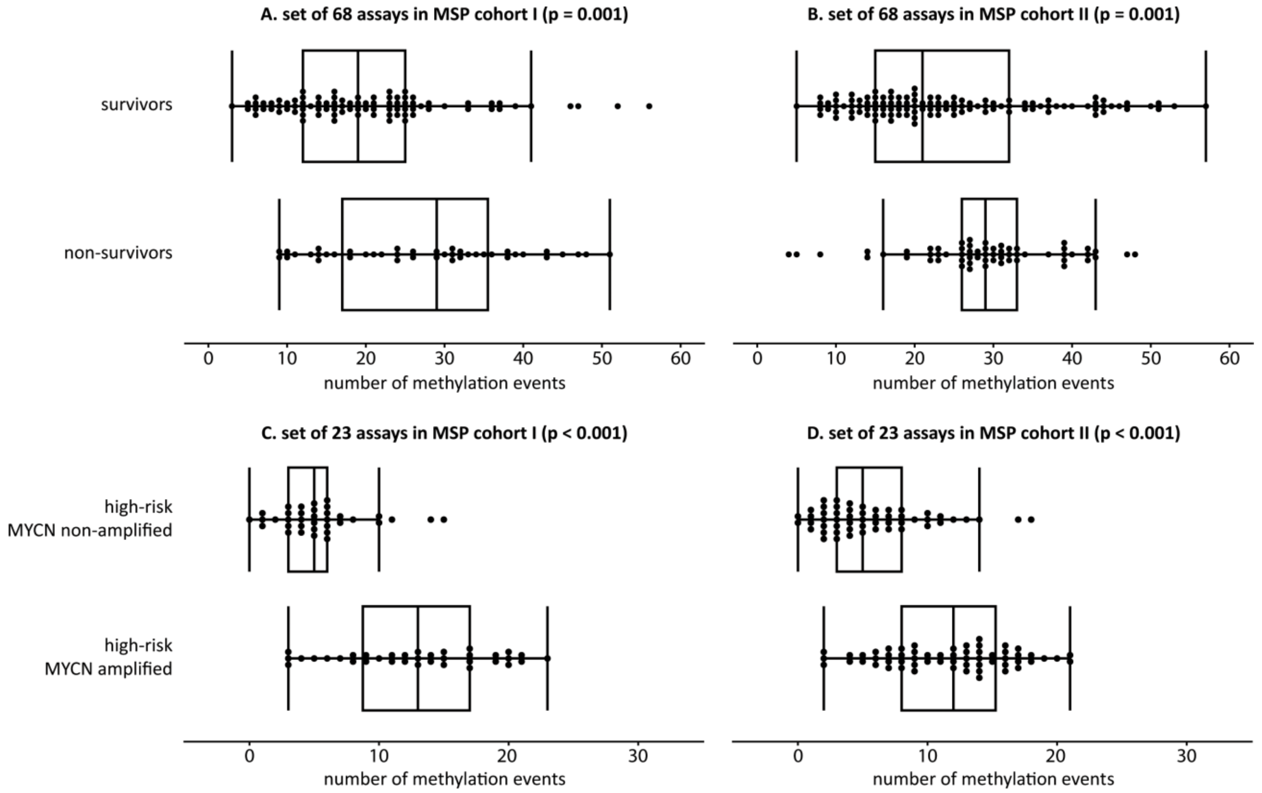

Supplemental figure 3. Methylation-specific PCR confirms the validity of methyl-CpG-binding domain sequencing in identifying candidate methylation markers. Number of methylation events of 68 MSP assays (designed in regions identified in the MBD sequencing data as being hypermethylated in non-survivors) in survivors (LR-SURV and HR-SURV) and non-survivors of MSP cohort I (**A.**; Mann-Whitney, $p = 0.001$) and MSP cohort II (**B.**; Mann-Whitney, $p = 0.001$). Number of methylation events of 23 MSP assays (designed in regions identified in the MBD sequencing data as being hypermethylated in high-risk *MYCN* amplified samples) in high-risk *MYCN* non-amplified and amplified samples of MSP cohort I (**C.**; Mann-Whitney, $p < 0.001$) and MSP cohort II (**D.**; Mann-Whitney, $p < 0.001$).

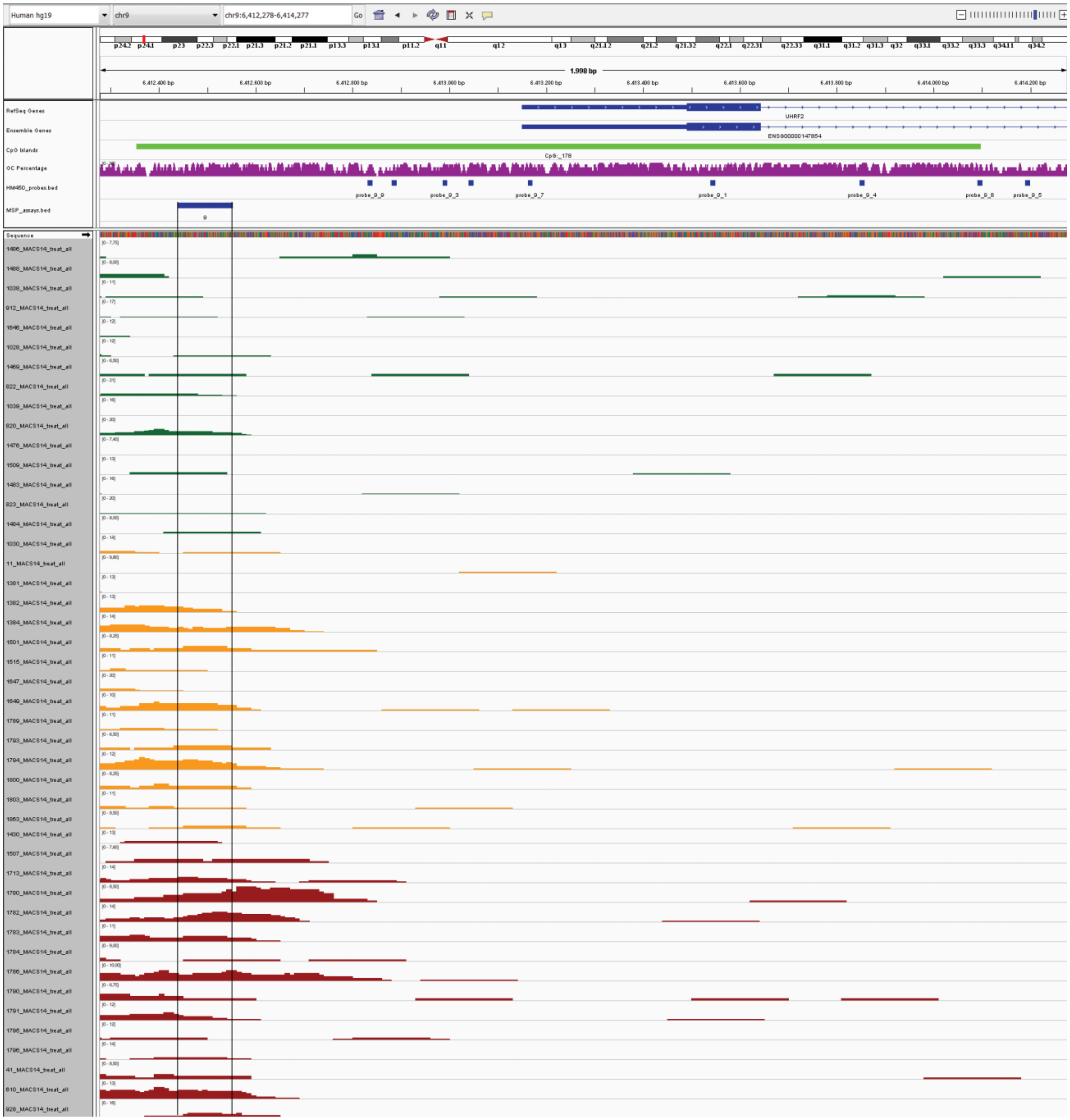

Human hg19
chr9
chr9:6,412,278-6,414,277
Go
1,998 bp
RefSeq Genes
Ensemble Genes
CpG Islands
GC Percentage
HM450_probes.bed
MSP_assays.bed
UHRF2
Sequence

Supplemental figure 4. Visualization of the MBD sequencing data of the promoter region of *UHRF2* shows that the corresponding MSP assay would not have been identified using the HM450 array. The location of the in-house designed MSP assay (large blue bar in upper panel) and probes of the HM450 array (small blue bars in the upper panel) are shown, as well as the number of sequencing tags at each position for each primary tumor of MBD cohort II (lower panel). High-risk non-survivors are indicated in red, high-risk survivors in orange and low-risk survivors in green.

Supplemental Table 1. In total, 437 annotated primary neuroblastoma DNA samples were collected and assigned to a specific study subcohort. A. Detailed characteristics. Each sample is characterized by a unique patientID and is assigned to a prognostic risk group (LR-SURV, HR-SURV or HR-DOD) and subcohort (MBD cohort I, MBD cohort II, MSP cohort I or MSP cohort II). Clinical characteristics given are the age at diagnosis in months, International Neuroblastoma Staging System (INSS) stage, *MYCN* amplification status (0 is non-amplified and 1 is amplified), and overall survival (OS) and event-free survival (EFS) status and time after diagnosis in days. The OS status indicates whether the patient was alive (0) at the last known follow-up or died of disease (1). Similarly, the EFS status indicates events such as relapse, progression or death. NAs represent missing values. LR-SURV: low-risk survivors, HR-DOD: high-risk deceased patients, HR-SURV: high-risk survivors. **B.** Summary. Per subcohort an overview of the clinical characteristics is given. [*]Only samples with a positive *ACTB* call were included, as only these were used in the analyses.

Supplemental Table 2. Seventy-eight MSP assays were designed, technically validated and tested for overall and event-free survival prediction. For each assay, if available, the gene annotation is shown, as well as the forward and reverse primer (5' to 3'), and the genomic location of the amplicon on the hg19 reference genome. The corresponding region of interest in the methyl-CpG-binding domain sequencing data and the comparisons in which the region was identified as differentially methylated (indicated by yes, followed by the group that is hypermethylated) are indicated, as well as log-rank p-values for overall and event-free survival in the corresponding test cohort. Furthermore, it is shown whether the assay is part of the 58-marker signature or not.

Supplemental Table 3. Nineteen neuroblastoma cell lines are included in the study. For each cell line, the *MYCN* amplification status is shown, as well as its corresponding number in the MSP screen.

Supplemental Table 4. The LabChip GX size and height, and LC480 Cq and Tm value were combined to construct a dichotomous calling matrix. A. Results on MSP cohort I. For each assay and sample, the methylation call (dark blue (1) is methylated, yellow (0) unmethylated) is given. The patient samples are subdivided into three prognostic groups (LR-SURV, HR-DOD and HR-SURV). LR-SURV: low-risk survivors, HR-DOD: high-risk deceased patients, HR-SURV: high-risk survivors, CL: cell line, U-HCT: negative control (HCT-116 DKO cell line), M-HCT: positive control (*in vitro* methylated HCT-116 DKO cell line) and NTC: no template control. **B.** Results on MSP cohort II. **C.** Summary on MSP cohort I and MSP cohort II (only *ACTB* positive samples are taken into account). The number and percentage of methylated samples for a particular MSP assay within each prognostic group and for the entire sample cohorts is given.

Supplemental Table 5. The 58-marker methylation signature is an independent prognostic predictor of overall survival in MSP cohort II. For each variable, the p-value, odds ratio (OR) and 95% confidence interval (CI) of the univariable and multivariable logistic regression analyses are shown. The age at diagnosis cutoff is 18 months.
